# Liver Fibrosis as a Predictor of Cardiovascular Risk in Patients with Severe Obesity

**DOI:** 10.3390/jcm14238532

**Published:** 2025-12-01

**Authors:** Alina N. Saidi, Willy B. Theel, Vivian D. de Jong, Stefanie R. van Mil, Aart-Jan van der Lely, Diederick E. Grobbee, Jan Apers, Ellen van der Zwan-van Beek, Manuel Castro Cabezas

**Affiliations:** 1 Department of Internal Medicine, Franciscus Gasthuis & Vlietland, 3045 PM Rotterdam, The Netherlands; m.castrocabezas@franciscus.nl; 2 Department of Internal Medicine, Erasmus University Medical Center, 3015 GD Rotterdam, The Netherlands; w.theel@erasmusmc.nl (W.B.T.); s.vanmil@erasmusmc.nl (S.R.v.M.); a.vanderlelij@erasmusmc.nl (A.-J.v.d.L.); 3 Julius Center for Health Science and Primary Care, University Medical Center Utrecht, 3584 CG Utrecht, The Netherlands; vivian.dejong@juliusclinical.com (V.D.d.J.); diederick.grobbee@juliusclinical.com (D.E.G.); 4 Julius Clinical, 3704 EC Zeist, The Netherlands; 5 Department of Surgery, Franciscus Gasthuis & Vlietland, 3045 PM Rotterdam, The Netherlands; j.apers@franciscus.nl; 6 Department of Clinical Chemistry, Franciscus Gasthuis & Vlietland, 3045 PM Rotterdam, The Netherlands; e.vanderzwan-vanbeek@franciscus.nl

**Keywords:** MASLD, MASH, FIB-4, obesity, liver fibrosis

## Abstract

**Background:** Obesity is a substantial global health issue associated with increased risk of cardiovascular disease (CVD) and metabolic dysfunction-associated steatotic liver disease (MASLD). Despite the known link between obesity, CVD and MASLD, it remains unknown which factors contribute to higher cardiovascular (CV) risk in patients with obesity-induced liver fibrosis. Liver fibrosis, assessed by the Fibrosis-4 (FIB-4) index, may help to identify patients with obesity at increased CV risk. **Methods:** Patients with severe obesity (Body Mass Index (BMI) ≥ 40 kg/m^2^) scheduled for bariatric surgery were subdivided into FIB-4 categories. Systemic leukocyte activation markers were measured by flow cytometry. Additionally, markers of vascular damage, namely the carotid intima media thickness (cIMT) and pulse wave velocity (PWV), were included. **Results:** The cohort predominantly consisted of women (74%) with an average age of 41 years and mean BMI of 42.7 kg/m^2^. Patients with an elevated FIB-4 (≥1.3) had higher systolic (146 ± 16 vs. 139 ± 15, *p* = 0.002) and diastolic blood pressure (91 ± 13 vs. 83 ± 12, *p* = 0.002), increased cIMT (0.66 ± 0.11 vs. 0.55 ± 0.10, *p* < 0.001), and higher PWV (8.2 ± 0.9 vs. 6.8 ± 1.1, *p* < 0.001) compared to those with a low FIB-4 (<1.3). Additionally, patients with a high FIB-4 tended to show increased expression of CD66b on granulocytes. **Conclusions:** Patients with severe obesity who were at risk of liver fibrosis showed greater signs of vascular damage, insulin resistance, and systemic inflammation. This suggests that liver fibrosis can be a useful marker for identifying patients with obesity at high CV risk.

## 1. Introduction

Obesity has emerged as a significant global health problem, with its prevalence steadily rising in recent decades [1,2]. According to the World Health Organization (WHO), more than 2.5 billion adults with overweight were reported in 2022, of which over 890 million were classified as people with obesity [3]. This increasing trend has significant implications for public health, given the numerous clinical complications associated with obesity, including cardiovascular disease (CVD), which remains the leading cause of morbidity and mortality worldwide, particularly among individuals with obesity [4].

Besides CVD, obesity is also strongly related to metabolic dysfunction-associated steatotic liver disease (MASLD), formerly known as non-alcoholic fatty liver disease (NAFLD), a spectrum of liver diseases ranging from simple steatosis, in the presence of at least one cardiometabolic risk factor, to more severe stages such as metabolic dysfunction-associated steatohepatitis (MASH), which is characterized by steatosis, hepatocyte ballooning, and lobular inflammation with or without fibrosis, cirrhosis, and hepatocellular carcinoma (HCC) [5,6,7,8,9]. The prevalence of MASLD among individuals with obesity is notably high and is expected to rise alongside the global obesity rates [10]. Population studies indicate that MASLD can be found in 65% of patients with obesity (BMI 30–39.9 kg/m^2^) and in approximately 85% of patients with severe obesity (BMI ≥ 40 kg/m^2^) [11].

While obesity is closely linked to both CVD and MASLD, the factors determining individual susceptibility to cardiovascular (CV) complications remain unclear. Liver fibrosis, which can develop as a consequence of chronic inflammation in obesity, may be associated with an elevated CV risk. Excessive fat accumulation in adipose tissue drives chronic inflammation in obesity. This metabolic stress leads adipocytes to release adipokines. These signaling molecules recruit and activate innate immune cells, including granulocytes and monocytes [12,13,14]. Upon activation, these cells express surface receptors like CD66b and CD11b. The increased expression of CD66b, in particular, serves as a reliable marker for neutrophil activation [15]. Once activated, immune cells release pro-inflammatory cytokines and chemokines, which promote endothelial dysfunction and further perpetuate the inflammatory state in obesity [16,17]. This chronic inflammation also facilitates the infiltration of leukocytes into the liver, contributing to the development of liver fibrosis [18]. Given the role of liver fibrosis in increasing CV risk, its presence in individuals with obesity may play a critical role in determining their higher susceptibility to CV complications. This study focuses on patients with severe obesity, a high-risk group that remains underrepresented in prior liver fibrosis research. Our hypothesis is that the presence of liver fibrosis, as indicated by the Fibrosis-4 (FIB-4) index, may distinguish patients with severe obesity at higher CV risk from their counterparts. The FIB-4 index is a non-invasive, cost-effective tool that estimates the degree of liver fibrosis and serves as an alternative to liver biopsy, which is invasive and carries potential risks [19,20].

This study aims to investigate the association between FIB-4 and CV risk factors in patients with severe obesity, and to evaluate the potential contribution of systemic leukocyte activation markers to liver fibrosis and CV risk.

## 2. Materials and Methods

### 2.1. Study Design

This study is a post hoc analysis of the ASSISI project, which was a prospective study with the primary aim to evaluate the effects of bariatric surgery on complement component 3 (C3) and systemic inflammatory markers associated with CV risk in patients with severe obesity [21,22,23]. The study protocol was in accordance with the Declaration of Helsinki, approved by the Medical Ethical Committee Rotterdam (Maasstad Hospital, Rotterdam, the Netherlands; ABR no. NL47891.101.14, approval date: 29 April 2015) and registered in the Dutch Trial register (NTR5172) [24]. Prior to collecting data written informed consent was obtained from each participant.

### 2.2. Study Population

The study population consisted of 200 patients aged 18 to 65 who were scheduled for bariatric surgery. All patients met the criteria of the International Federation for the Surgery of Obesity (IFSO) for bariatric surgery [25]. Inclusion criteria were: having a body mass index (BMI) of ≥40 kg/m^2^, or a BMI of 35 kg/m^2^ and obesity related clinical complications, including hypertension, type 2 diabetes mellitus (T2DM), dyslipidemia, respiratory disorders, severe joint disease, and significant obesity-related psychological or functional limitations. Patients were excluded if they were using immune-modulating medication, if they had an acute inflammatory disease within 6 weeks prior to inclusion, or if they previously underwent bariatric surgery or cholecystectomy. All data were collected at study entry, prior to the bariatric procedure.

### 2.3. Baseline Characteristics

Baseline characteristics were obtained during standard preoperative screening prior to bariatric surgery and included medical history and current medication profile. Anthropometric measurements included waist circumference, height, weight, and blood pressure. BMI was calculated using weight and height.

### 2.4. Laboratory Measurements

Preoperative laboratory tests were performed on non-fasting blood samples following the hospital’s standard procedures [22]. Leukocyte activation markers (CD11b and CD66b) were measured by flow cytometry using blood samples collected in EDTA tubes. Fluorescent-labeled antibodies against CD66b and CD11b were used to identify granulocytes and monocytes, with CD45 used to distinguish leukocytes from other blood cells. Whole blood was incubated with antibodies for 15 min at room temperature in the dark, with isotype controls included to correct for nonspecific binding. Erythrocytes were lysed using a standard isotonic solution. Samples were analyzed using a Coulter Epics XL-MCL (Beckman Coulter, Miami, FL, USA) or Navios flow cytometer (Beckman Coulter, Miami, FL, USA), and data were processed with EXPO 32 or Kaluza 1.2 software. Marker expression was reported as mean fluorescence intensity in arbitrary units [26].

### 2.5. Carotid Intima Media Thickness

Carotid intima media thickness (cIMT) measurements were performed by a trained and experienced sonographer using the ART-LAB (Esaote, Genoa, Italy) following procedures outlined in prior studies [22,27]. Ultrasound scans were performed with participants in a supine position, head resting comfortably and neck slightly hyperextended and rotated opposite to the probe. Images of the distal 1 cm of the far wall of each common carotid artery (CCA) were obtained using B-mode ultrasound, producing two echogenic lines representing the combined thickness of the intima and media layers. Each CCA was imaged in three views (right side: 90°, 120°, 150°; left side: 210°, 240°, 270°), and the segments were measured semi-automatically in triplicate. The mean of these measurements was used for analyses.

### 2.6. Pulse Wave Velocity

Pulse wave velocity (PWV) was measured using the Mobil-O-Graph (I.E.M. Germany) [28]. To measure the PWV, an inflatable cuff was placed on the patient’s bare left upper arm, with the cuff size chosen based on arm circumference. These cuffs detect fluctuations in cuff pressure caused by pulse waves when inflated to sub-diastolic pressures [29]. PWV is calculated by analyzing the time delay between these fluctuations at different arterial locations. This value reflects the speed at which a mechanical wave propagates along the arterial wall, serving as an indicator of arterial stiffness. Higher PWV values are linked to increased vascular stiffness, which is a significant predictor of CV events [30]. To ensure accuracy and reliability, PWV measurements were performed in triplicate.

### 2.7. Fibrosis-4 Index

The FIB-4 is the most commonly used non-invasive test (NIT) to estimate the degree of hepatic fibrosis. The FIB-4 was calculated according to the following formula: $FIB-4=\frac{Age\left(years\right)\times AST(U/L)}{Platelet\;count\left(10^{9}/L\right)\times \sqrt{ALT}\;(U/L)}$. Based on previously published literature, a cut-off of 1.3 was applied, as values ≥ 1.3 have been associated with an increased risk of advanced fibrosis [7,31]. Low fibrosis risk was defined as a FIB-4 < 1.3 and high fibrosis risk as a FIB-4 ≥ 1.3.

### 2.8. Statistical Analyses

All statistical analyses were conducted using IBM SPSS statistics version 28.0.1.0 (IBM SPSS Statistics, New York, NY, USA). Continuous variables are presented as mean ± SD or median (IQR), and categorical variables as frequencies (%). Patients were stratified into a low FIB-4 group (<1.3) and a high FIB-4 group (≥1.3) based on the EASL recommendation on NITs [7,32,33]. Between-group differences (low vs. high FIB-4) were assessed using independent samples *t*-tests or Mann–Whitney U tests as appropriate. Associations between medication use and FIB-4 categories were evaluated using Chi-square tests. Correlation analyses were performed using Pearson’s correlation or Spearman’s correlation where applicable. The primary aim of this post hoc analysis was to examine FIB-4 in relation to vascular and metabolic parameters. Established relationships between classical cardiometabolic risk factors and subclinical vascular measures in this cohort have been reported previously [22,28]. Associations between FIB-4 and vascular or metabolic parameters were assessed using multivariable regression analyses. For continuous FIB-4, a linear regression model included key demographic and cardiometabolic variables (age, cIMT, sex, BMI, systolic BP, HbA1c, LDL-C, HDL-C, triglycerides, creatinine, CRP). For dichotomized FIB-4 (<1.3 vs. ≥1.3), an exploratory logistic regression was applied using the same set of predictors (*N* = 92 with complete data). To account for potential non-linear effects of age, a separate sensitivity analysis modeled age using four linear spline terms (knots at 30, 40, and 50 years). Systemic leukocyte activation markers were included as exploratory outcomes, and no formal correction for multiple testing was applied. *p*-values of <0.05 were considered statistically significant.

## 3. Results

**Figure 1:**
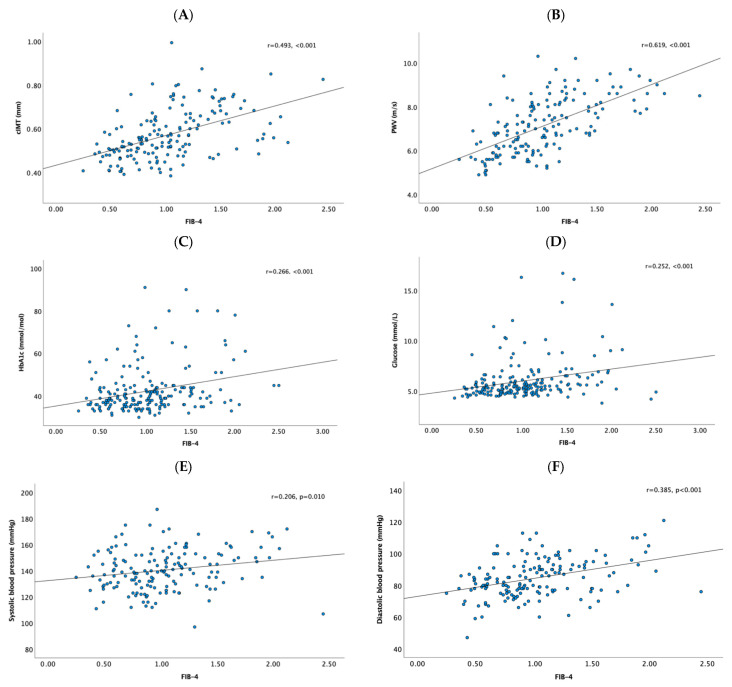
Correlations of the Fibrosis-4 (FIB-4) index with (**A**) carotid intima-media thickness (cIMT), (**B**) pulse wave velocity, (**C**) glycated hemoglobin A1c (HbA1c), (**D**) glucose levels, (**E**) systolic blood pressure, and (**F**) diastolic blood pressure.

**Figure 2:**
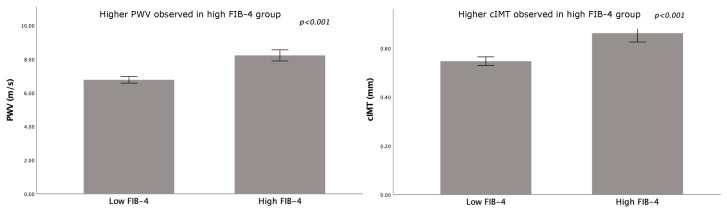
Mean pulse wave velocity (PWV) and carotid intima-media thickness (cIMT) values by FIB-4 group. Low FIB-4 (<1.3) vs. high FIB-4 (≥1.3): 6.8 ± 1.1 vs. 8.2 ± 0.9 m/s for PWV, and 0.55 ± 0.10 vs. 0.66 ± 0.11 mm for cIMT. Error bars indicate standard error of the mean (SE).

The total cohort consisted of 200 patients, predominantly women (*N* = 148), with a mean age of 41 ± 12 years and a mean BMI of 42.7 ± 5.2 kg/m^2^. FIB-4 values in the study population ranged from 0.23 to 2.5, with a mean of 1.02 ± 0.44 (Table A1). Of the total cohort, 155 patients (77.5%) were classified as having a low FIB-4 score (<1.3), while 45 patients (22.5%) had a high FIB-4 score (≥1.3). Additional details can be found in Table A1. BMI and waist circumference were not significantly different between patients with low and high FIB-4 scores. The group with high FIB-4 scores was older (Table 1). Patients in the elevated FIB-4 group showed higher systolic and diastolic blood pressure compared to those with low FIB-4. They were also more frequently treated with antihypertensive medications (48.9% vs. 27.7%, *p* = 0.011) (Table 1). Significant positive correlations were observed between FIB-4 and both systolic and diastolic blood pressure (Figure 1). Additionally, those with a high FIB-4 showed increased cIMT and PWV, with positive correlations observed between FIB-4 and both cIMT and PWV (Figure 2). Moreover, those with a high FIB-4 showed increased levels of HbA1c and glucose (Table 1), with correlation analyses indicating significant positive associations between FIB-4 and both HbA1c and glucose levels (Figure 1). Classical CVD risk factors such as low HDL-C, high LDL-C, and high TG did not show significant differences between the groups (Table 1). Subgroup analyses compared lipid levels in patients based on hypertension, statin use, and T2DM status. Overall, no major differences in lipid levels were observed between low and high FIB-4 across these subgroups. Patients in the high FIB-4 group showed higher use of statins, antihypertensive medications, and antidiabetic medication, including the use of insulin (Table 2). Those in the high FIB-4 group also tended to have an increased expression of granulocyte CD66b (Table 3), with a significant positive correlation between FIB-4 and granulocyte CD66b (rho = 0.177, *p* = 0.012). Conversely, these patients tended to have a decreased expression of monocyte CD11b surface markers (Table 3). Subgroup analyses (Table A2) showed that hypertensive patients with high FIB-4 had significantly higher cIMT and PWV than those with low FIB-4 (cIMT: 0.67 ± 0.11 vs. 0.59 ± 0.11 mm; PWV: 8.4 ± 0.9 vs. 7.4 ± 1.0 m/s; both *p* < 0.001), consistent with greater arterial stiffness. In diabetic patients, cIMT and PWV were numerically higher in the high FIB-4 group, but differences were not statistically significant (*p* = 0.29 and *p* = 0.07). Multivariable regression of baseline demographics and cardiometabolic factors, showed that only age and cIMT were independently associated with continuous FIB-4 (Table A3). Multivariable logistic regression of dichotomized FIB-4 (low vs. high) including 92 patients with complete data showed that age and HbA1c were independent predictors, whereas cIMT and CRP showed trends but were not significant (Table A4). Multivariable regression analysis identified age as the most significant predictor of cIMT (*p* < 0.001). CD66b on granulocytes also significantly influenced cIMT (*p* = 0.033), indicating its role in cIMT variation. Sex showed a significant negative association with cIMT (*p* = 0.003), with women exhibiting lower cIMT values than men. FIB-4 categories approached significance (*p* = 0.069), suggesting a potential, though less clear, link to cIMT. No significant associations were observed between cIMT and BMI, systolic blood pressure, or diastolic blood pressure (Table 4). To account for potential non-linear effects of age, age was modeled using four linear spline terms with knots at 30, 40, and 50 years. In this spline-adjusted model, FIB-4 remained significantly associated with cIMT (B = 0.054, *p* = 0.026), whereas the age spline segments were not statistically significant (Table A5).

## 4. Discussion

This study evaluated liver fibrosis, assessed by FIB-4, in patients with severe obesity and its association with CV risk markers, including cIMT and PWV. Patients with elevated FIB-4 exhibited early vascular changes, higher blood pressure, insulin resistance (IR), and systemic inflammation. Traditional CV risk factors, including BMI and lipid profiles, neither differentiated between groups nor showed independent associations with cIMT. Regression analyses revealed that sex and granulocyte CD66b expression were independently associated with cIMT, whereas FIB-4 showed a borderline association that became significant in spline-adjusted models accounting for age. Women had lower cIMT values, emphasizing sex as an important modifier of early vascular alterations. These results suggest that classical measures may have limited predictive value for early subclinical atherosclerosis in this population, which appears to be primarily mediated by fibrosis and inflammatory pathways.

Analyses treating FIB-4 as a continuous variable showed significant associations with age and cIMT, while exploratory logistic regression using dichotomized FIB-4 (≥1.3) revealed associations with age and HbA1c, with cIMT and CRP showing non-significant trends. The smaller number of individuals above the FIB-4 threshold likely reduced statistical power in the logistic analysis.

Patients with elevated FIB-4 had higher systolic and diastolic blood pressure despite more frequent use of antihypertensive medications, suggesting a greater burden of hypertension or CV comorbidity. This is consistent with the higher prevalence of hypertension in MASLD [34,35] and a cross-sectional study of 4705 patients showing an association between hypertension and liver fibrosis (OR 2.0, 95% CI 1.3–3.0), which was stronger in obesity (OR 2.1, 95% CI 1.4–3.2) [36]. Both hypertensive and non-hypertensive subgroups also showed that higher FIB-4 was associated with increased cIMT and PWV, reflecting greater arterial stiffness. These observations are reinforced by external cohorts, where in 8336 adults higher PWV was independently associated with MASLD [37], and in 2550 participants advanced liver fibrosis nearly doubled the risk of elevated cIMT and increased PWV [38], further underscoring the link between liver fibrosis and vascular dysfunction.

Metabolic factors further distinguished patients with elevated FIB-4. Glucose and HbA1c were elevated independently of BMI and despite more intensive antidiabetic therapy, highlighting the central role of IR and glycemic dysregulation in liver fibrosis. Most diabetic patients in the overall cohort had FIB-4 < 1.3, consistent with prior studies [39,40], but T2DM was relatively more frequent among patients with elevated FIB-4, suggesting a link between glycemic status and fibrosis burden that warrants further investigation in larger cohorts.

Despite greater use of statins and other cardiometabolic medications, vascular damage in patients with elevated FIB-4 was not mitigated. While statins can induce vascular calcification, this generally reflects plaque stabilization rather than increased CV risk, and randomized trials demonstrate that statins reduce atherosclerotic progression [41,42,43]. These findings imply that FIB-4 identifies individuals with severe obesity who remain at residual CV risk despite intensive preventive therapy.

Inflammatory profiling revealed higher CD66b expression on granulocytes in patients with elevated FIB-4, whereas CD11b expression on monocytes was decreased. This pattern suggests a shift toward granulocyte-driven inflammation, while monocyte-mediated pathways, typically involved in atherosclerosis [44,45], appear less prominent. These results align with prior studies showing granulocyte activation contributes to systemic inflammation and vascular alterations in liver disease [46,47].

Lipid profiles did not differ significantly between groups, likely due to the higher prevalence of statin use in patients with elevated FIB-4. This observation is consistent with the understanding that advanced liver disease can reduce total cholesterol and LDL-C via impaired hepatic cholesterol synthesis, whereas statin therapy may mask underlying dyslipidemia [46,47,48,49]. Importantly, fibrosis has been linked to CV risk independent of lipid abnormalities [50,51].

FIB-4 was selected for its widespread validation, guideline endorsement, and ease of clinical application [7]. Although other non-invasive fibrosis scores, such as the NAFLD Fibrosis Score, are available, elevated FIB-4 effectively identifies patients with severe obesity at high CV risk. Future studies should assess how FIB-4 compares with alternative scores in predicting CV outcomes, which may further refine risk stratification in this population.

Collectively, these findings suggest that targeting liver fibrosis could prevent early vascular alterations independently of traditional risk factors, while granulocyte activation may mechanistically link liver fibrosis to vascular dysfunction. FIB-4 may identify patients with persistent CV risk despite optimal therapy, highlighting its potential as a clinically useful biomarker beyond traditional markers such as BMI, blood pressure, and lipid profiles. Given the exploratory nature of the study, these results are hypothesis-generating, and provide a basis for future larger, well-designed studies investigating the mechanisms linking liver fibrosis, inflammation, sex differences, and early CVD in this population.

The current study has several limitations. The study population was predominantly female, reflecting a common trend in research involving severe obesity. The relatively small, exploratory, hypothesis-generating design limited the scope of analyses and precluded exhaustive multivariable modeling. Non-fasting blood samples may have introduced variability but reflect real-world clinical conditions. Only a limited number of inflammatory markers were analyzed, and follow-up FIB-4 measurements or imaging-based assessments (e.g., ultrasound or elastography) were not available. Medication use was reported descriptively, without adjustment for confounding by indication, which could be addressed in larger, future studies. Importantly, this post hoc analysis evaluated multiple vascular outcomes without a pre-specified primary endpoint or formal sample size calculations, yet it provides novel, hypothesis-generating insights into the links between liver fibrosis, systemic inflammation, and subclinical CVD in bariatric surgery candidates.

## 5. Conclusions

In conclusion, assessing liver fibrosis in individuals with severe obesity can help identify those with a very high CV risk. Our findings suggest that incorporating FIB-4 into routine CV risk assessment could improve patient stratification and support more tailored preventive strategies in this high-risk population.

## Figures and Tables

**Table 1:** Baseline characteristics by FIB-4 group: comparison of low FIB-4 (<1.3) versus high FIB-4 (≥1.3). Data presented as mean (standard deviation), median (interquartile range), or number (%).

	Low FIB-4 N = 155	High FIB-4 N = 45	*p*-Value
Demographic characteristics			
Age (years)	38 ± 11	53 ± 5.2	<0.001
Sex, n (%) female	123 (79)	25 (56)	0.001 *
Body weight (kg)	124 ± 19	126 ± 28	0.691
Body mass index (kg/m^2^)	42.5 ± 4.4	43.3 ± 7.2	0.458
Waist circumference (cm)	129 ± 12	131 ± 16	0.341
Diabetes mellitus, n (%)	27 (17.4)	16 (35.6)	0.013 ^†^
Clinical characteristics			
FIB-4	0.83 ± 0.26	1.67 ± 0.30	<0.001
Hematocrit (L/L)	0.42 ± 0.04	0.43 ± 0.03	0.524
Hemoglobin (mmol/L)	8.7 ± 0.8	8.8 ± 0.7	0.194
Systolic blood pressure (mmHg)	139 ± 15	146 ± 16	0.002
Diastolic blood pressure (mmHg)	83 ± 12	91 ± 13	0.002
Total cholesterol (mmol/L)	5.1 ± 1.0	5.4 ± 1.2	0.197
Triglycerides (g/L)	2.0 ± 1.1	2.2 ± 1.2	0.359
HDL-C (mmol/L)	1.2 ± 0.29	1.3 ± 0.31	0.278
LDL-C (mmol/L)	3.1 ± 0.92	3.3 ± 1.1	0.221
Apolipoprotein B (g/L)	1.1 ± 0.29	1.2 ± 0.33	0.069
HbA1c (mmol/mol)	41 ± 9.5	48 ± 14	0.002
Glucose (mmol/L)	5.7 ± 1.6	7.0 ± 2.9	0.006
Insulin (µIU/mL)	23 (17–44)	30 (18–47)	0.998
C-reactive protein (mg/L)	6 (3–10)	5 (3–9)	0.199
Creatinine (µmol/L)	65 ± 14	71 ± 16	0.038
Homocystein (µmol/L)	11.5 ± 4.1	14.0 ± 9.6	0.167
TSH (mU/l)	1.8 (1.4–2.3)	2.0 (1.5–2.6)	0.934
Medication			
Statins, n (%)	16 (10.3%)	15 (33.3%)	<0.001
Antihypertensives, n (%)	43 (27.7%)	22 (48.9%)	0.011
Antidiabetics, n (%)	22 (14.2%)	13 (28.9%)	0.027

Abbreviations: FIB-4, Fibrosis-4 index; HDL-C, high-density lipoprotein cholesterol; LDL-C, low-density lipoprotein cholesterol; HbA1c, hemoglobin A1c; TSH, thyroid stimulating hormone. * *p*-value for sex calculated using Pearson Chi-square. ^†^ Forty patients had a prior diagnosis of T2DM, and three were newly diagnosed at the time of inclusion.

**Table 2:** Chi-square test results for the association between low FIB-4 (<1.3) versus high FIB-4 (≥1.3) and medication use.

Medication	Low FIB-4 (%)	High FIB-4 (%)	X^2^	* p * -Value
Statins	10.3	33.3	14.099	<0.001
Antihypertensives	27.7	48.9	7.109	0.011
Antidiabetics	14.2	28.9	5.217	0.027

**Table 3:** Flow cytometry results for leukocyte activation markers by FIB-4 group (low < 1.3 vs. high ≥ 1.3).

Leukocyte Activation Marker	Low FIB-4	High FIB-4	*p*-Value
CD35 on monocytes (au)	7.3 ± 3.6	7.1 ± 3.0	0.680
CD35 on granulocytes (au)	10.7 ± 5.0	10.5 ± 6.5	0.815
CD11b on monocytes (au)	34.6 ± 10.2	31.6 ± 7.8	0.070
CD11b on granulocytes (au)	47.5 ± 15	44.8 ± 16	0.303
CD66b on granulocytes (au)	5.9 ± 1.8	6.7 ± 2.6	0.070

**Table 4:** Multivariable linear regression analysis of variables associated with carotid intima media thickness (cIMT).

	Standardized Coefficients Beta	VIF	*p*-Value
(Constant)	-	0.016	-
Age (years)	0.438	1.782	<0.001
CD66b on granulocytes (au)	0.163	1.356	0.033
CD11b on monocytes (au)	0.061	1.427	0.429
FIB-4	0.143	1.443	0.069
Sex (m/f)	−0.229	1.339	0.003
BMI (kg/m^2^)	0.063	1.241	0.387
Systolic blood pressure (mmHg)	0.039	2.267	0.691
Diastolic blood pressure (mmHg)	−0.033	2.576	0.749

Abbreviations: FIB-4; Fibrosis-4 index; BMI, Body Mass Index; VIF, Variance Inflation Factor.

## Data Availability

The original contributions presented in this study are included in the article. Further inquiries can be directed to the corresponding author.

## Abbreviations

The following abbreviations are used in this manuscript:

ALT	Alanine aminotransferase
AST	Aspartate aminotransferase
cIMT	Carotid intima media thickness
CV	Cardiovascular
CVD	Cardiovascular disease
FIB-4	Fibrosis-4 index
HCC	Hepatocellular carcinoma
IFSO	International Federation for the Surgery of Obesity
MASLD	Metabolic dysfunction-associated steatotic liver disease
MASH	Metabolic dysfunction-associated steatohepatitis
NAFLD	Non-alcoholic fatty liver disease
NIT	Non-invasive test
PWV	Pulse wave velocity
T2DM	Type 2 diabetes mellitus
WHO	World health organization

## Appendix A

**Table A1:** Baseline clinical and demographic characteristics of the total cohort (*N* = 200). Data are given as mean (standard deviation), median (interquartile range) or number (%).

	Patients (N = 200)
Demographic characteristics	
Sex (f/m) [n, %]	148 (74)/52 (26)
Age (years)	41 ± 12
Body weight (kg)	125 ± 21
Body mass index (kg/m^2^)	42.7 ± 5.2
Ethnicity Caucasian [n, %]	145 (73.2)
African [n, %]	20 (10.1)
Latin-American [n, %]	6 (3.0)
Indian [n, %]	8 (4.0)
Asian [n, %]	1 (0.5)
Clinical characteristics	
Hematocrit (L/L)	0.43 ± 0.04
Hemoglobin (mmol/L)	8.7 ± 0.81
Systolic blood pressure (mmHg)	140 ± 16
Diastolic blood pressure (mmHg)	85 ± 12
Total cholesterol (mmol/L)	5.2 ± 1.1
LDL-C (mmol/L)	3.1 ± 0.96
HDL-C (mmol/L)	1.2 ± 0.29
Triglycerides (mmol/L)	1.7 (1.2–2.7)
Apolipoprotein B (g/L)	1.1 ± 0.3
Glucose (mmol/L)	6.1 ± 2.4
C-reactive protein (mg/L)	6 (3.0–10)
Leukocytes (10^9^/L)	8.7 ± 2.4
Carotid Intima Media thickness (mm)	0.57 ± 0.11
Medication	
Statins [n, %]	31 (15.5)
Antihypertensives [n, %]	65 (32.5)
Antidiabetics [n, %]	35 (17.5)
Medical history	
Diabetes [n, %]	43 (21.5)
Hypertension [n, %]	69 (34.5)
Hypercholesterolemia [n, %]	58 (29)
CVA or TIA [n, %]	5 (2.5)
Myocardial infarction	6 (3)
Fibrosis markers	
Fibrosis-4 index (FIB-4)	
Total population	1.02 ± 0.44
FIB-4 score <1.3 [n, %]	155 (77.5)
FIB-4 score ≥1.3 [n, %]	45 (22.5)

Abbreviations: HDL-C, high-density lipoprotein cholesterol; LDL-C, low-density lipoprotein cholesterol; CVA, cerebrovascular accident; TIA, transient ischemic attack; FIB-4, Fibrosis-4 index.

**Table A2:** Subgroup analyses of cIMT and PWV according to FIB-4 category in hypertensive and diabetic patients.

Subgroup	FIB-4 Category	cIMT (mm)	N	*p*-Value cIMT	PWV (m/s)	N	*p*-Value PWV
Hypertensive	Low FIB-4	0.59 ± 0.11	66	<0.001	7.4 ± 1.0	64	<0.001
	High FIB-4	0.67 ± 0.11	28		8.4 ± 0.9	25	
Diabetic	Low FIB-4	0.60 ± 0.11	21	0.303	7.4 ± 1.0	20	0.079
	High FIB-4	0.64 ± 0.10	10		8.2 ± 0.9	8	

Notes: Values are mean ± SD. *p*-values compare low vs. high FIB-4 within each subgroup. Abbreviations: cIMT, carotid intima media thickness; PWV, pulse wave velocity; FIB-4, Fibrosis-4 index.

**Table A3:** Multivariable linear regression of factors associated with continuous FIB-4.

Variable	B	Std. Error	Beta	*p*-Value	VIF
Constant	−0.566	0.660	-	0.394	-
Age (years)	0.021	0.004	0.501	<0.001	2.02
cIMT (mm)	0.832	0.416	0.208	0.049	2.04
Sex (female/male)	−0.130	0.100	−0.126	0.198	1.77
BMI (kg/m^2^)	0.007	0.008	0.083	0.373	1.62
Systolic BP (mmHg)	−0.001	0.003	−0.018	0.831	1.35
HbA1c (mmol/mol)	0.005	0.004	0.105	0.215	1.32
LDL-c (mmol/L)	0.017	0.037	0.038	0.651	1.28
HDL-c (mmol/L)	0.029	0.172	0.017	0.865	1.80
Triglycerides (g/L)	0.008	0.048	0.014	0.877	1.63
Creatinine (µmol/L)	−0.002	0.004	−0.036	0.695	1.60
CRP (mg/L)	−0.007	0.005	−0.118	0.162	1.30

Notes: Dependent variable: FIB-4. VIF values indicate no substantial multicollinearity among included variables. Abbreviations: FIB-4, Fibrosis-4 index; cIMT, carotid intima media thickness; BMI, body mass index; BP, blood pressure; HbA1c, glycated hemoglobin; LDL-c, low-density lipoprotein cholesterol; HDL-c, high-density lipoprotein cholesterol; CRP, C-reactive protein; VIF, Variance Inflation Factor.

**Table A4:** Multivariable logistic regression of factors associated with dichotomized FIB-4 (<1.3 vs. ≥1.3).

Predictor	OR	95% CI	*p*-Value
Age (years)	1.266	1.089–1.471	0.002
cIMT (per 0.1 mm)	1.947	0.871–4.354	0.107
Sex (female/male)	0.339	0.040–2.881	0.363
BMI (kg/m^2^)	1.064	0.907–1.247	0.446
Systolic BP (mmHg)	1.011	0.960–1.065	0.692
HbA1c (mmol/mol)	1.100	1.000–1.210	0.049
LDL-C (mmol/L)	1.597	0.614–4.150	0.336
HDL-C (mmol/L)	1.902	0.116–31.198	0.758
Creatinine (µmol/L)	1.404	0.482–4.089	0.522
CRP (mg/L)	0.990	0.909–1.078	0.838

Note: cIMT was rescaled per 0.1 mm to provide interpretable odds ratios. Abbreviations: OR, odds ratio; CI, confidence interval; cIMT, carotid intima media thickness; BMI, body mass index; BP, blood pressure; HbA1c, glycated hemoglobin; LDL-C, low-density lipoprotein cholesterol; HDL-C, high-density lipoprotein cholesterol; CRP, C-reactive protein.

**Table A5:** Regression of carotid intima-media thickness (cIMT) on FIB-4 and age modeled with splines.

Predictor	B	Standard Error	*p*-Value
Constant	0.431	0.088	<0.001
FIB-4	0.054	0.024	0.026
Age spline 1	0.001	0.003	0.839
Age spline 2	0.006	0.003	0.066
Age spline 3	0.004	0.003	0.166
Age spline 4	0.004	0.004	0.264

Note: Dependent variable: carotid intima-media thickness (cIMT). Age was modeled using four linear spline terms with knots at 30, 40, and 50 years (Age spline 1–4).
